# Quality Improvement in Acute Ischemic Stroke Care in Taiwan: The Breakthrough Collaborative in Stroke

**DOI:** 10.1371/journal.pone.0160426

**Published:** 2016-08-03

**Authors:** Fang-I Hsieh, Jiann-Shing Jeng, Chang-Ming Chern, Tsong-Hai Lee, Sung-Chun Tang, Li-Kai Tsai, Hsun-Hsiang Liao, Hang Chang, Kenneth A. LaBresh, Hung-Jung Lin, Hung-Yi Chiou, Hou-Chang Chiu, Li-Ming Lien, for the BTS-Stroke Investigators

**Affiliations:** 1 School of Public Health, Taipei Medical University, Taipei, Taiwan; 2 Stroke Center and Department of Neurology, National Taiwan University Hospital, Taipei, Taiwan; 3 Neurological Institute, Taipei Veterans General Hospital, Taipei, Taiwan; 4 Department of Neurology, Chang Gung Memorial Hospital and College of Medicine, Chang Gung University, Linkou, Taiwan; 5 Taiwan Joint Commission on Hospital Accreditation, Taipei, Taiwan; 6 RTI International, Waltham, Massachusetts, United States of America; 7 Department of Emergency Medicine, Chi-Mei Medical Center, Tainan, Taiwan; 8 Department of Biotechnology, Southern Taiwan University of Science and Technology, Tainan, Taiwan; 9 Department of Neurology, Shin Kong Wu Ho-Su Memorial Hospital, Taipei, Taiwan; 10 College of Medicine, Fu Jen Catholic University, Taipei, Taiwan; 11 School of Medicine, College of Medicine, Taipei Medical University, Taipei, Taiwan; Massachusetts General Hospital, UNITED STATES

## Abstract

In the management of acute ischemic stroke, guideline adherence is often suboptimal, particularly for intravenous thrombolysis or anticoagulation for atrial fibrillation. We sought to improve stroke care quality via a collaborative model, the Breakthrough Series (BTS)-Stroke activity, in a nationwide, multi-center activity in Taiwan. A BTS Collaborative, a short-term learning system for a large number of multidisciplinary teams from hospitals, was applied to enhance acute ischemic stroke care quality. Twenty-four hospitals participated in and submitted data for this stroke quality improvement campaign in 2010–2011. Totally, 14 stroke quality measures, adopted from the Get With The Guideline (GWTG)-Stroke program, were used to evaluate the performance and outcome of the ischemic stroke patients. Data for a one-year period from 24 hospitals with 13,181 acute ischemic stroke patients were analyzed. In 14 hospitals, most stroke quality measures improved significantly during the BTS-activity compared with a pre-BTS-Stroke activity period (2006–08). The rate of intravenous thrombolysis increased from 1.2% to 4.6%, door-to-needle time ≤60 minutes improved from 7.1% to 50.8%, symptomatic hemorrhage after intravenous thrombolysis decreased from 11.0% to 5.6%, and anticoagulation therapy for atrial fibrillation increased from 32.1% to 64.1%. The yearly composite measures of five stroke quality measures revealed significant improvements from 2006 to 2011 (75% to 86.3%, p<0.001). The quarterly composite measures also improved significantly during the BTS-Stroke activity. In conclusion, a BTS collaborative model is associated with improved guideline adherence for patients with acute ischemic stroke. GWTG-Stroke recommendations can be successfully applied in countries besides the United States.

## Introduction

It has been estimated that 16 million people suffer a stroke each year worldwide, and that 5.7 million will die as a result; moreover, around half of stroke survivors will suffer permanent disability [1]. Stroke is the leading cause of serious long-term disability in adults, resulting in significant disability-adjusted life year loss [2,3]. Strategies to decrease disability, improve functional outcomes, and prevent stroke recurrence are key aspects of effective acute stroke care. Indeed, many evidence-based stroke care guidelines have been updated in accordance with these considerations to successfully guide clinical practice procedures for stroke or transient ischemic attack [4–8]. However, with an adherence rate to evidence-based care of only 55% [9], a significant gap still exists between recommended guidelines and actual clinical practice. Adherence to evidence-based guidelines for treatment of stroke or transient ischemic attack is often suboptimal [10,11], and the availability of published recommended clinical guidelines is not in itself sufficient for successful management. Strategies that incorporate educational programs, as well as quality surveys on the implementation of the guidelines in clinical practice, are needed to improve stroke care quality [12].

Stroke performance measures (or stroke metrics) are important for quantifying improvements in the quality of stroke care [13]. Previously, several stroke performance measures have been applied in stroke care quality improvement programs, including the Primary Stroke Center and Comprehensive Stroke Center designation, the Center for Disease Control and Prevention Paul Coverdell National Acute Stroke Registry, the Get With The Guidelines (GWTG)-Stroke program in the United States [14–18], the Danish National Indicator Project [19], the Netherlands Stroke Survey [20], and the Catalonia Stroke Program [21]. The Taiwan Stroke Registry (TSR), undertaken from 2006 to 2008, revealed that the GWTG-Stroke performance measures are applicable in Taiwan, and that several performance measures needed to be improved to achieve a standard of care consistent with the GWTG-Stroke program [22]. In accomplishing this, the TSR study emphasized the importance of thrombolytic therapy, as well as anticoagulant treatment for atrial fibrillation (AF) and use of lipid lowering drugs for low density lipoprotein (LDL) ≥100 mg/dL [22].

The Institute for Healthcare Improvement, which seeks to improve health care by supporting process change, conceptualized the Breakthrough Series (BTS)–a model for achieving breakthrough improvements in quality while reducing costs—in 1994 [23,24]. A BTS collaboration is a short-term learning system that brings together a large number of teams from hospitals in order to drive improvements on a focused topic. The present study aimed to demonstrate the improved quality of acute ischemic stroke management via a BTS collaborative learning model in a nationwide, multi-center activity in Taiwan.

## Methods

### The Taiwan BTS-Stroke activity

Since 2006, the Taiwan Joint Commission on Hospital Accreditation has adopted the BTS model to improve healthcare quality in Taiwan for several focused topics. Improvement of acute stroke care quality was the main concern of the Taiwan Joint Commission on Hospital Accreditation in 2010. In May and June of 2010, an expert panel of neurologists, neurosurgeons, emergency medicine specialists, and stroke nursing specialists identified 14 quality measures for effective management and prevention of acute ischemic stroke. A BTS-Stroke activity was conducted from August 2010 to July 2011, in which hospitals with interests in acute stroke care quality improvement were encouraged to participate. Over a one-year period, the BTS-Stroke activity consisted of three learning sessions and a final summative meeting. For each hospital team, three to seven members could attend the learning sessions. The activity followed the key elements of the BTS model, including topic selection, faculty recruitment, enrollment of participating organizations and teams, learning sessions, action periods, improvement of the model, summative congresses and publications, and measurement and evaluation [23,24].

#### Data collection

Data used to calculate the BTS-Stroke quality and safety measures were compiled from medical record by trained neurologists and their study nurses or stroke case managers. The definitions of each quality and safety measures were clearly written in the proposal and explained to each participating hospitals in learning sessions. The number of eligible patients and the number of interventions performed were reported to the BTS data center every month from August 2010 to July 2011. Therefore, there were no personal details in all data reported to BTS data center, only the name of hospital was available. Before the data released for statistical analysis, a code number was given instead of the hospital’s name. In this study, ethics approval with waiver of informed consent was provided by the Institutional Review Board of Taipei Medical University.

#### BTS-Stroke quality measures

Nine GWTG-Stroke quality measures and one safety measure were used to assess the quality of acute ischemic stroke care and prevention [18]: (1) intravenous tissue plasminogen activator (IV tPA) use in patients who arrived at a participating hospital <2 hours from symptom onset; (2) antithrombotic medication use within 48 hours of admission; (3) discharge prescription of antithrombotics; (4) discharge prescription of oral anticoagulants for AF; (5) prescription of lipid lowering drugs at discharge for LDL ≥100 mg/dL; (6) time of door-to-computed tomography (CT) ≤25 minutes in patients presenting with stroke symptoms of <3 hours duration; (7) dysphagia screening prior to any oral intake; (8) assessment for stroke rehabilitation services; (9) stroke education provided to patient and/or caregiver; and (10) symptomatic intracerebral hemorrhage (ICH) after IV tPA treatment. In addition, four quality measures were also included: (1) time of door-to-needle (DTN) ≤60 minutes for patients who arrived <2 hours from symptom onset; (2) percentage of acute ischemic stroke patients that underwent IV tPA treatment; (3) frequency (%) of intra-arterial thrombolysis for all acute ischemic stroke patients; and (4) 30-day mortality for all stroke patients. For non-ambulatory patients, we did not include anticoagulant prophylaxis for deep venous thrombosis within 48 hours of admission as a quality measure, because symptomatic deep venous thrombosis is rare in immobilized patients including those with stroke in Asia [25]. Moreover, deep venous thrombosis only occurred in 0.2% of patients in the TSR study performed from 2006 to 2008 [22]. The topic of smoking cessation was included in stroke education. Symptomatic ICH after IV tPA treatment was defined as any ICH event documented by CT or MRI within 36 hours of IV tPA therapy, with National Institutes of Health Stroke Scale (NIHSS) deterioration of two or more points. DTN time ≤60 minutes were included because a decrease in the in-hospital delay can improve functional outcome after IV tPA treatment. In addition, the Target: Stroke initiative of the American Heart Association/American Stroke Association, proposed in 2011, aimed to achieve DTN time ≤60 minutes in more than 50% of patients [26].

#### Statistical analysis

In Tables 1 and 2, categorical variables were presented as percentages, and either chi-square test or Fisher’s exact test, as appropriate, was used for comparisons between the two groups or study periods. Composite measures, similar to those reported in the GWTG-Stroke program in USA, were also derived based on five individual performance measures. The composite score was defined as the total number of interventions performed among eligible patients divided by the total number of possible interventions among eligible patients. In Table 3, the composite score reflects a summary score of performance of all five individual performance measures. However, the composite score comprises 11 performance measures except IA thrombolysis, symptomatic ICH after IV tPA, and 30-day mortality in Table 4. The multivariate linear regression model adjusting for hospital types was used to test the trends of quality measures and the composite score from 2006 to 2011 in Table 3 and quarterly composite performance measures in Table 4. Statistical significance was set at a two-sided *p* value <0.05. Analyses were performed using SAS statistical software version 9.1 (SAS Institute, Cary, NC).

**Table 1:** Quality Measures of 24 Hospitals Participating in BTS-Stroke Activity in 2010–2011.

	Overall	Group I	Group II	*P* value
**Hospital no.**	24	14	10	
**Hospital types**				
Medical center	33.3%	50%	10%	0.079
Regional hospital	66.7%	50%	90%	
**Bed size** (median, 25th–75th percentile)	621.5 (428, 784.5)	664 (499, 799)	600.5 (334, 736)	
≧ 621.5 beds		57.1%	40.0%	0.680
**Patient no.**	13,181	7,492	5,689	
**Measures**				
Door to CT ≤25 minutes	77.2%	76.1%	78.9%	0.134
IV tPA <2 hours	79.5%	80.0%	78.7%	0.473
IV tPA percentage	4.1%	4.6%	3.4%	0.171
Door to needle ≤60 minutes	52.8%	50.8%	56.7%	0.008
Symptomatic ICH after IV tPA	6.0%	5.6%	6.7%	0.306
IA thrombolysis percentage	0.5%	0.9%	0%	0.008
Dysphagia screening	76.1%	74.9%	77.8%	0.127
Early antithrombotics	96.6%	96.8%	96.4%	0.622
Anticoagulants for AF	57.1%	64.1%	48.0%	<0.001
Lipid lowering drugs for LDL ≥100 mg/dL	63.4%	68.0%	57.3%	<0.001
Antithrombotics at discharge	94.0%	94.6%	93.3%	0.223
Rehabilitation evaluation	68.1%	79.5%	55.6%	<0.001
Stroke education	91.9%	94.9%	88.5%	<0.001
30-day mortality	3.5%	4.1%	2.8%	0.110

Group I, hospitals participated in the Taiwan Stroke Registry in 2006–2008; Group II, hospitals did not participate in the Taiwan Stroke Registry in 2006–2008. AF, atrial fibrillation; CT, computed tomography; IA, intraarterial; ICH, intracerebral hemorrhage; IV tPA, intravenous tissue plasminogen activator; LDL, low-density lipoprotein.

**Table 2:** Comparison of Quality Measures from 14 Hospitals in Group I Before and During BTS-Stroke Activity and the GWTG-Stroke in USA (2003–2009).

	GWTG-Stroke	Before BTS	During BTS	*P* value
**Period**	2003/4~2009/8	2006/5~2008/7	2010/8~2011/7	
**Patient no.**	601,599	9,612	7,492	
**Measures**				
Door to CT ≤25 minutes	35.6%	33.9%	76.1%	<0.001
IV tPA <2 hours	59.5%	16.3%	80.0%	<0.001
IV tPA percentage	4.3%	1.2%	4.6%	<0.001
Door to needle ≤60 minutes	26.6%	7.1%	50.8%	<0.001
Symptomatic ICH after IV tPA	5.4%	11.0%	5.6%	<0.001
IA thrombolysis percentage	--	0.1%	0.9%	0.026
Dysphagia screening	68.3%	5.6%	74.9%	<0.001
Early antithrombotics	95.1%	93.7%	96.8%	0.001
Anticoagulants for AF	91.1%	32.1%	64.1%	<0.001
Lipid lowering drugs for LDL ≥100 mg/dL	77.5%	38.2%	68.0%	<0.001
Antithrombotics at discharge	95.9%	85.5%	94.6%	<0.001
Rehabilitation evaluation	95.4%	--	79.5%	--
Stroke education	72.3%	--	94.9%	--
30-day mortality	5.5%^a^	4.2%	4.1%	0.914

AF, atrial fibrillation; CT, computed tomography; IA, intraarterial; ICH, intracerebral hemorrhage; IV tPA, intravenous tissue plasminogen activator; LDL, low-density lipoprotein.

^a^ In-hospital mortality.

**Table 3:** Trends of Quality Measures in 14 Hospitals in Group I Before and During BTS-Stroke Activity (2006–2011).

	2006	2007	2008	2010	2011	Trend test^a^
						β (SE), *p* value
**Performance measures**						
IV tPA <2 hours	18.0	15.2	17.1	85.3	77.6	16.5 (1.5), <0.001
Early antithrombotics	91.8	94.3	95.8	97.1	96.6	0.4 (0.3), 0.178
Antithrombotics at discharge	85.5	85.3	87.8	92.5	95.9	1.8 (0.4), <0.001
Anticoagulation for AF	34.7	30.7	34.4	64.7	63.9	4.2 (1.1), <0.001
Lipid-lowering drug for LDL ≥100 mg/dL	37.1	40.2	43.6	64.5	70.3	6.3 (1.0), <0.001
Composite measure	75.0±4.5	74.0±4.5	74.6±4.2	88.4±6.8	86.3±9.2	2.9 (0.5), <0.001
**Safety measure**						
Symptomatic ICH after IV tPA	9.7	13.2	8.0	6.5	4.4	-1.4 (1.3), 0.278

Values are percentage except for composite measure (mean±std).

IV tPA, intravenous tissue plasminogen activator; AF, atrial fibrillation; LDL, low-density lipoprotein; ICH, intracerebral hemorrhage.

^a^ Adjusted for hospital types.

**Table 4:** Quarterly Composite Performance Measures of 24 Hospitals Participating in BTS-Stroke.

	Q1	Q2	Q3	Q4	Overall	Trend test^b^
Performance measures^a^	mean±std	mean±std	mean±std	mean±std	mean±std	β (SE), *p* value
All hospitals (n = 24)	63.12±11.03	66.06±9.68	67.61±10.35	69.25±7.39	66.82±8.34	2.00 (0.85), 0.0203
Group I hospitals (n = 14)	66.05±11.39	67.05±12.21	68.02±13.25	69.69±9.11	68.14±10.31	1.19 (1.31), 0.3691
Group II hospitals (n = 10)	59.01±9.56	64.67±4.53	67.04±4.43	68.63±4.34	64.98±4.19	3.12 (0.87), 0.0009

Group I, hospitals participated in the Taiwan Stroke Registry in 2006–2008; Group II, hospitals did not participate in the Taiwan Stroke Registry in 2006–2008.

^a^ Comprising 11 performance measures (IA thrombolysis, symptomatic ICH after IV tPA, and 30-day mortality are not included).

^b^ Adjusted for hospital types.

## Results

Data collected from 24 hospitals over a one-year period for 13,181 patients with acute ischemic stroke were analyzed. Fourteen of the 24 hospitals had been involved (to varying extents) in the TSR study conducted between 2006 and 2008, while 10 had not. Fifty percent of TSR hospitals (Group I) were medical centers and the median value of bed size was 664. Ninety percent of non-TSR hospitals (Group II) were regional hospitals and the median value of bed size was 600.5. The results of the stroke quality measures for all of the hospitals, as well as a comparison between the 14 TSR hospitals (*n* = 7,492) and the 10 non-TSR hospitals (*n* = 5,689) are shown in Table 1. Compared to non-TSR hospitals, TSR hospitals had a higher frequency of performing IA thrombolysis (0.9% vs. 0%, p = 0.008), more frequent prescriptions of oral anticoagulants for AF patients (64.1% vs. 48.0%, p<0.001), and more frequent prescriptions of lipid lowering drugs for patients with LDL ≥100 mg/dL (68.0% vs. 57.3%, p<0.001). In addition, rehabilitation evaluation (79.5% vs. 55.6%, p<0.001) and stroke education (94.9% vs. 88.5%, p<0.001) were more frequently used in TSR hospitals than that in non-TSR hospitals. However, DTN time ≤ 60 minutes were less frequent in TSR hospitals (50.8% vs. 56.7%, p = 0.008). The reason may be that half of TSR hospitals are medical centers, a DTN time ≤60 minutes may not be achievable in all ischemic stroke patients treated in medical centers, particularly those with unstable hemodynamics, respiratory compromise, or challenging clinical presentations.

For the 14 hospitals that participated in both the TSR study (2006–2008) and the present BTS-Stroke collaboration (2010–2011), a comparison of stroke quality measures was made between the two periods (Table 2). The results from the GWTG-Stroke program in USA are also listed for comparison. We found that most stroke quality measures were improved significantly. The rate of IV tPA use increased from 1.2% to 4.6% (p<0.001), DTN time ≤60 minutes improved from 7.1% to 50.8% (p<0.001), symptomatic hemorrhage after IV tPA decreased from 11.0% to 5.6% (p<0.001), anticoagulation treatment for AF increased from 32.1% to 64.1% (p<0.001), and lipid lowering drugs for LDL ≥100 mg/dL increased from 38.2% to 68% (p<0.001). The rates of IV tPA use, symptomatic ICH after IV tPA, early antithrombotics, antithrombotics at discharge during BTS -Stroke Activity were comparable to GWTG-Stroke in USA.

Yearly individual and composite measures of five stroke quality indicators, i.e. IV tPA <2 hours, early antithrombotic use, antithrombotic prescription at discharge, anticoagulation treatment for AF, and use of lipid lowering drugs for LDL ≥100 mg/dL, are shown in Table 3. Except for early antithrombotic use, significant improvements were observed for all measures and composite measures from 2006 to 2011. The percentage of IV tPA <2 hours dramatically improved from 18% to 77.6% between 2006 and 2011. The percentage of anticoagulation for AF raised from 34.7% to 63.9% and the use of lipid-lowering drug for LDL ≥ 100 mg/dL increased from 37.1% to 70.3%.

The quarterly changes in the composite scores of 11 performance measures—excluding intraarterial thrombolysis, symptomatic ICH after IV tPA, and 30-day mortality for all patients—are shown in Table 4 for the participating hospitals during the BTS-Stroke activity. A significant trend for overall quality improvement was found from 63.12 to 69.25 during the Q1-Q4 period (β = 2.00±0.85, p = 0.0203), particularly for non-TSR hospitals (β = 3.12±0.87, p = 0.0009).

## Discussion

The present study showed that quality of acute stroke care can be improved significantly after a well-organized, multi-center stroke quality improvement activity. The campaign activity described in this study was conducted through the BTS model introduced by the Institute for Healthcare Improvement in 1994 [23,24]. The BTS model has been implemented in previous studies of health-related topics. In the present study, we applied the BTS model in order to improve the quality of acute ischemic stroke care. The BTS activity consisted of three learning sessions and one final summative meeting. During each learning session, several multidisciplinary teams (each coming from separate institutions) met with each other, alongside subject experts, to communicate their experiences and to discuss the barriers they faced in their clinical practice. In this way, participants exchanged knowledge and reflected on strategies for improvement of the various measures. Associated with this one-year activity, improvement in 10 of 13 processes of acute ischemic stroke care was found, with highly significant improvements in thrombolytic therapy, dysphagia screening, anticoagulation treatment for AF, and prescription of lipid lowering drugs for LDL ≥100 mg/dL.

We put an emphasis on thrombolytic therapy for acute ischemic stroke patients because six of our 14 quality measures were related to thrombolytic therapy. Through the BTS-Stroke activity, the rate for IV tPA use was significantly increased with nearly four-fold from 1.2% in 2006–08 to 4.6% in 2010–11, which was equivalent to the GWTG-Stroke program. To highlight the importance of reducing the in-hospital delay for IV tPA administration, we selected DTN time ≤60 minutes as a quality measure. In the GWTG-Stroke program, less than 30% of acute ischemic stroke patients treated with IV tPA had DTN times ≤60 minutes (19.5% in 2003 to 29.1% in 2009) [27]. The guideline recommendation specifies that IV tPA treatment should be initiated within 60 minutes of arrival for acute ischemic patients (if there is no contraindication) [4]. During the BTS-Stroke learning sessions, hospitals with records of good performance for IV tPA use demonstrated best practice strategies to improve DTN time (consistent with the key strategies chosen by the Target: Stroke initiative) [26]. Many strategies, including pre-hospital notification, rapid triage, computerized stroke code, high priority of CT performance, and point-of-care testing, were widely adopted by the participating hospitals [26,28–30]. The proportion of patients with DTN ≤60 minutes increased significantly from 7.1% in 2006–08 (before BTS) to 50.1% in 2010–11 (during BTS). This was accompanied by a decreased rate of symptomatic ICH (11.0% in 2006–08 to 5.6% in 2010–11).

Our previous TSR results also showed that only one-third of patients with AF were prescribed oral anticoagulants at discharge [22]. In a population study of AF patients in Taiwan, only 38.9% were in adherence with the 2006 American Heart Association/American College of Cardiology and the European Society of Cardiology (AHA/ACC/ESC) recommendations for antithrombotic therapy; among high risk patients, this was reduced to 24.4% (due in large part to underuse of oral anticoagulants) [31]. Both physicians and patients may have concerns regarding the increased risk of ICH associated with anticoagulants use; indeed, anticoagulants-related ICH was shown to occur more frequently in Asians than in other race/ethnicities (hazard ratio for ICH with whites as referent was 4.06 for Asians) [32]. However, through careful monitoring and frequent evaluation, these risks can be minimized. A computerized alarm reminder system for prescription at discharge was proposed by some hospitals as a way of improving adherence to quality measures. Due to the BTS-Stroke activity, the rate of oral anticoagulants use in eligible AF patients increased from 32.1% to 64.1%, which was still lower than that in the GWTG-Stroke program (91.1%) [33].

The risks of pneumonia and mortality are increased if acute stroke patients have dysphagia. Dysphagia screening can significantly decrease stroke-associated pneumonia, resulting in better functional outcomes [34,35]. Prior to this BTS activity, little formal dysphagia screening was being conducted in acute stroke patients in Taiwanese hospitals. In the TSR study from 2006 to 2008, only 5.6% patients were screened for swallowing function before their first oral intake. During the BTS-Stroke activity, one stroke nursing expert educated the participating teams on the necessity for dysphagia screening and provided details on its implementation [36]. Thereafter, adherence to dysphagia screening showed a marked improvement to 74.9%, similar to the GWTG-Stroke program (68.3%).

Taiwan is the first country outside of the United States to apply the quality measures of the GWTG-Stroke program, through evaluation of quality of acute stroke management in hospitals that participated in our previous TSR study [22]. The results highlighted a significant gap compared with the GWTG-Stroke program and guideline recommended targets for acute ischemic stroke patients were often suboptimal, especially in the areas of intravenous thrombolytic therapy, warfarin treatment for AF, and use of lipid lowering drugs for LDL ≥100 mg/dL. It is noteworthy that the purpose of TSR study was to establish a reliable stroke database for assessing acute stroke care quality and identifying areas that require improvement. There was no mandate from government for improvement in acute stoke care quality over time in TSR and quality improvement strategies were not included. While the Taiwan BTS-Stroke activity, conducted by the Taiwan Joint Commission on Hospital Accreditation, focus on the improvement of acute stroke care quality by group learning and competition in 2010. This is the reason why in Table 3, the significant trends of quality improvement for most indicators over the study period are attributed to the abrupt improvement between 2008 and 2010 rather than a gradual improvement. For hospitals participating in the TSR in 2006–2008, they were more familiar to their weak points in acute stoke care quality by reviewing their TSR data. Therefore they may improve quickly during the Q1 period in Taiwan BTS-Stroke activity. For non-TSR hospitals, the obvious improvements were observed during Q1-Q3 periods. These may partially explain the reason why the trend of improvement in composite performance measure during the BTS-activity for the hospitals participating in the previous TSR was not significant whereas it was significant among the hospitals without a previous experience of TSR in Table 4. A recent study reported successful adoption of GWTG-Stroke measures by a Brazilian tertiary hospital for quality improvement [37]. Several large-scale stroke registries that used stroke performance measures for quality evaluation indicated that systematic collection and evaluation of stroke performance measures can lead to rapid improvement in stroke care [38]. Our experience indicates that the BTS activity [23,24], – a multi-disciplinary, collaborative learning and feedback model—can further improve the quality of stroke care.

Our study has some limitations. Firstly, not all hospitals that participated in the previous TSR study were included in the present BTS-Stroke campaign activity (only 14 of the 39 TSR hospitals are represented in the present study). Nevertheless, this activity revealed many practical and effective strategies that can be transferred to other hospitals. The TSR continues to monitor the quality of acute stroke care according to the established quality measures. The Taiwan Joint Commission on Hospital Accreditation will implement the Taiwan Clinical Performance Indicator on stroke, mainly based on the BTS-Stroke quality measures. Secondly, the BTS-Stroke activity did not store data for individual patients, and only analyzed results for the performance of each hospital. Therefore, it was not possible to adjust for important variables such as age, sex, and stroke severity. However, we have compared performance before and during the BTS activity in the same set of 14 hospitals, which minimizes the unadjusted bias. Thirdly, this is an observational study without a contemporary control group. Thus it is not possible to fully attribute the improvement in measure performance solely to the BTS activity. However a prior analysis from the GWTG-Stroke program assessed the relationship of calendar time and time in the GWTG-Stroke quality improvement program and demonstrated an independent association between time participating in the program and measure improvement [18]. Fourthly, current stroke quality measures, including our own, are often confined to admissions of acute stroke cases and the associated process measures [38]. Revised quality measures for other types of stroke (e.g. hemorrhagic stroke) and measures for long-term care are also needed.

In conclusion, a BTS collaborative learning and campaign model can improve the guideline adherence for management of acute ischemic stroke. The GWTG-Stroke recommendations can be successfully conducted outside of the United States.
